# Supplementary material for: The phased pan-genome of tetraploid European potato
Source: Nature. 2025 Apr 16;642(8067):389–97. doi: 10.1038/s41586-025-08843-0 (PMC12158759; doi:10.1038/s41586-025-08843-0)
Supplement: Supplementary file 5 — Supplementary Figs. 3–14, 20, 21, 23–27 and 34–44. [file 41586_2025_8843_MOESM5_ESM.zip › suppl_figure_21/suppl_figure_21b_chr02.pdf]

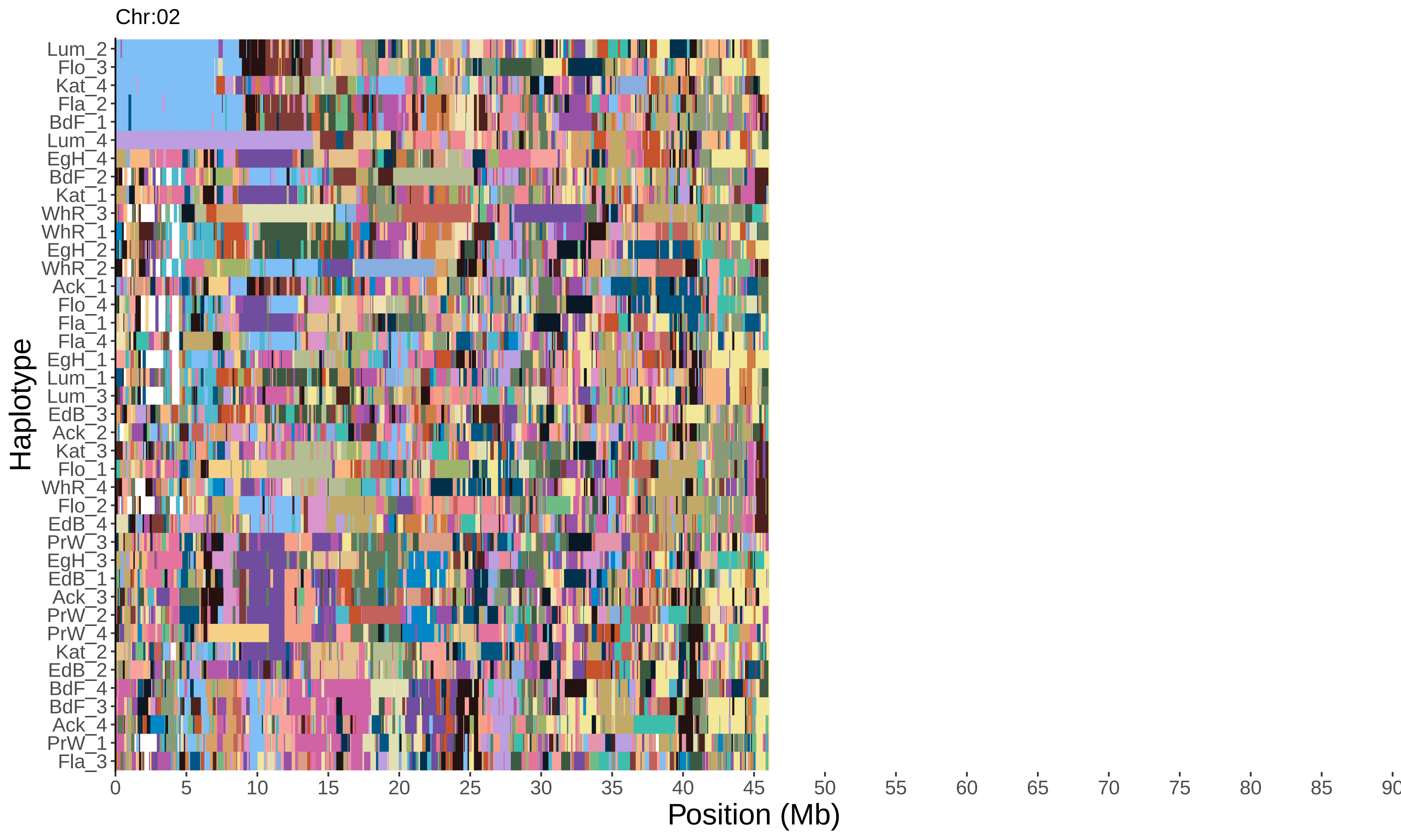
Chr:02
Lum_2
Flo_3
Kat_4
Fla_2
BdF_1
Lum_4
EgH_4
BdF_2
Kat_1
WhR_3
WhR_1
EgH_2
WhR_2
Ack_1
Flo_4
Fla_1
Fla_4
EgH_1
Lum_1
Lum_3
EdB_3
Ack_2
Kat_3
Flo_1
WhR_4
Flo_2
EdB_4
PrW_3
EgH_3
EdB_1
Ack_3
PrW_2
PrW_4
Kat_2
EdB_2
BdF_4
BdF_3
Ack_4
PrW_1
Fla_3
Haplotype
0 5 10 15 20 25 30 35 40 45 50 55 60 65 70 75 80 85 90
Position (Mb)
